# Self-Learning Methodology in Simulated Environments (MAES©) as a Learning Tool in Perioperative Nursing. An Evidence-Based Practice Model for Acquiring Clinical Safety Competencies

**DOI:** 10.3390/ijerph18157893

**Published:** 2021-07-26

**Authors:** Ester Peñataro-Pintado, José Luis Díaz-Agea, Isabel Castillo, César Leal-Costa, Antonio Jesús Ramos-Morcillo, María Ruzafa-Martínez, Encarna Rodríguez-Higueras

**Affiliations:** 1 Nursing Department, University School of Nursing and Occupational Therapy of Terrassa (EUIT), 08221 Terrassa, Spain; esterpenataro@euit.fdsll.cat; 2 Nursing Department, International University of Catalonia (UIC), Campus Sant Cugat, 08195 Sant Cugat del Vallès, Spain; icastillo@uic.es (I.C.); erodriguez@uic.es (E.R.-H.); 3 Nursing Department, Catholic University of Murcia (UCAM), 30107 Guadalupe de Maciascoque, Spain; 4 Nursing Department, University General Hospital of Catalonia (UIC), 08195 Sant Cugat del Vallès, Spain; 5 Nursing Department, University of Murcia, 30003 Murcia, Spain; cleal@um.es (C.L.-C.); maruzafa@um.es (M.R.-M.)

**Keywords:** self-directed learning, simulation, perioperative nursing training, MAES, evidence-based practice

## Abstract

Background: The self-learning Methodology in Simulated Environments (Spanish acronym: MAES©, (Murcia, Spain) is a type of self-directed and collaborative training in health sciences. The objective of the present study was to compare the level of competence of postgraduate surgical nursing students in the clinical safety of surgical patients, after training with the MAES© methodology versus traditional theoretical–practical workshops, at different points in time (post-intervention, after three months, six months post-intervention, and at the end of the clinical training period, specifically nine months post-intervention). Methods: We conducted a prospective study with an experimental group of surgical nursing postgraduate students who participated in MAES© high-fidelity simulation sessions, and a control group of postgraduate nursing students who attended traditional theoretical–practical sessions at two universities in Catalonia (Spain). The levels of competence were compared between the two groups and at different time points of the study. Results: The score was higher and statistically significantly different in the experimental group for all the competencies, with a large effect size at every measurement point previously mentioned. Conclusions: The postgraduate nurses were the most competent in the clinical safety of surgical patients when they trained with the MAES© methodology than when they learned through traditional theoretical–practical workshops. The learning of surgical safety competencies was more stable and superior in the experimental group who trained with MAES©, as compared to the control group.

## 1. Introduction

The great importance of clinical security was determined starting with the Institute of Medicine in the *To Err is Human: Building a Safety Health* System report [1], so that complications, human errors, or failures in the system ceased to occur. This report guided the quality policies of the main international health organization for the development of strategies and recommendations that promoted the control of inevitable damages. This became especially important in the field of surgery, due to the complexity and risks of the perioperative process [2,3], and this is what the World Health Organization (WHO) campaign” Safe Surgery Saves Lives” is based on.

The WHO provides important data to consider: up to 25% of surgical patients suffer from a complication, with a crude mortality rate of 0.5–5% after major surgery [4,5]. However, the most alarming data is that half of the adverse effects could have been avoided. The causes are diverse, but not following the safety principles or doing so irregularly or in an incomplete manner, is prominent [4,6,7]. This could also be due to diverse motives, such as a lack of motivation of the surgical team, not being convinced about the usefulness of the measure implemented, or a lack of training [8].

Therefore, the systematic promotion of safety dynamics in surgery by the institutions and the members that comprise the surgical team can save lives. Safety in the operating room is a complex task, but it is essential that the perioperative nurses understand and possess the skills necessary to guarantee that the patients are not exposed to errors. Providing safe care must be the main objective of every perioperative nurse [9,10].

To be able to develop safe and quality perioperative plans, it is necessary for nurses to receive specific training to acquire the necessary competencies associated with the safety of the surgical patient throughout the perioperative process. Additionally, that this training is based on evidence-based practices is also considered highly critical.

A recurring subject of debate among the education community is the style of teaching/learning that is most appropriate for the students to attain the corresponding competencies [11,12]. Many models have been described in the pedagogic and psychology literature [13]. The SimZones is a useful model [14] that is part of a recommendation for progressive learning with simulation, according to the level of fidelity and preparation of the participants. The model is a manner of structuring the simulation according to learning zones, which were described in 2017 in the context of the simulation program at the Boston Children’s Hospital. According to this approach, the simulations can be structured into four zones (Zones 0–3), with a progressive distribution in complexity and a number of competencies, distractions, and level of fidelity. Zones 0 and 1 are mainly focused on the acquisition of technical skills, and feedback is continuously provided by the facilitator. In Zone 2, more context is added so that the student acquires more decision-making competencies. A debriefing session takes place with the facilitator once the student finishes the task. Zone 3 allows for the development of non-technical skills, which are mainly focused on working with a multidisciplinary group. The participants are more skillful than in the previous zones, and the facilitator provides a debriefing session at the end of the scenario.

This approach is focused on programs developed in the hospital clinical environment, more so than in a university setting, but is presently becoming established in the nomenclature and classification of the organization of a simulation.

Zone 0 includes automatic feedback exercises for the students, who learn individually using virtual simulation technologies (problems, clinical cases, or computer programs designed specifically for training on technical abilities).

In Zone 1, the simulations require the participation of an instructor, with whom the students would practice their fundamental clinical skills without context and with little or no “noise” (probe, auscultate, inject, drain, suture, etc.).

In Zone 2, the simulations include situations with context (and increases in noise). The participants work in severe situations with the use of protocols (such as cardiopulmonary resuscitation, learning scenarios in clinical situations, etc.). They are normally students enrolled in health sciences degrees, those who train in a university center, or those who participate in a training course on life support, for example.

Simulations in Zone 3 imply a greater degree of realism and authenticity. The participants are real professionals who are training on their habitual competencies (native teams of participants, for example, real neonatal unit personnel who train with scenarios that are typical to their field and with actions that are very similar to what they do in their day-to-day).

Zone 4 has also been described as learning that can be achieved with group reflection (debriefing) after a real event experienced by a real work team.

The context of this study is based on a model of learning with a simulation where the role of the professor is modified, as he or she will have to take on a role as a motivator and guide in the learning process; more than just “teaching”, he or she facilitates “learning”.

The MAES© methodology (the Spanish acronym of “Metodología de Autoaprendizaje en Entornos Simulados”) was designed in 2013 and complies with all the INACSL (International Nursing Association of Clinical Simulation and Learning) Simulation Standards [15]. MAES© places the student at the center of the learning process by combining different learning models: self-directed learning [16] problem-based learning [17], collaborative learning [18], and peer education [19]. This method is organized into six sequential phases, divided between in-classroom work and out-of-classroom work, with a minimum of two in-person sessions.

With this method, the students work in teams and guide their learning. They design simulation scenarios according to their learning needs and their baseline level of competencies in subjects they have freely selected. Afterwards, they experience these simulation cases and discuss them with scientific evidence under the guidance of a facilitator.

The MAES© methodology develops comprehensive learning comprising various dimensions:

1. Self-directed and collaborative learning, as the students work in 2–3 person teams, design a scenario, and search for information about the selected case to share with their colleagues afterwards.
2. Experience-based learning, given that the students come into contact with a situation that is very close to reality, and this training conditions them so that they can appropriately respond to a clinical situation.
3. Reflective learning, as the participating students’ weak points are worked on, and their strong ones are strengthened. Through a structured debriefing, the participants reflect on the practice and a debate is established, which is very productive for learning. It should be highlighted that the autonomy of the students does not imply that the role of the facilitator is not important. In this sense, the facilitator is the pillar and the guide during the entire learning process.

Studies have been conducted that underline the benefits of the MAES methodology in the acquisition of the competencies in nursing degrees [20,21,22], as well as the good perception of the facilitators and students who have taken part in simulation sessions with MAES [23,24]. However, no experimental education studies (with a control group) with postgraduate nurses have been conducted on the impact of learning with this method.

To focus this study, the following research question was posed: Do postgraduate nurses perceive themselves as more competent in the clinical safety of the surgical patient when they are trained with the MAES© method than when they learn through traditional theoretical–practical workshops?

The aim of this study was to compare the level of competence in clinical safety of the surgical patient of postgraduate surgical nursing students after training with the MAES© methodology versus traditional theoretical–practical workshops at various points of time (post-intervention, after three months, six months post-intervention, and at the end of the clinical training period (nine months after the intervention)).

## 2. Materials and Methods

### 2.1. Design

A prospective study was conducted with an experimental group of postgraduate surgical nursing students who participated in the high-fidelity MAES© simulation sessions, and a control group composed of postgraduate nursing students who participated in traditional theoretical–practical sessions. The levels of competence were compared between the groups at different times: when finishing their training, after three months, after six months, and when the clinical practice of each group had ended (nine months after the intervention).

### 2.2. Participants

The sample was composed of 103 postgraduate students in surgical nursing from two universities in Spain (Universitat Internacional de Catalunya and the Escola Universitària d’Infermeria i Teràpia Ocupacional de Terrassa, ascribed to the Universitat Autònoma de Barcelona) in the 2018–2019 and 2019–2020 academic years. The recruitment took place at the start of the academic year, specifically in October 2018, and October 2019.

The inclusion criteria were that the participants were enrolled for the first time in the Surgical Nursing, Anesthesia, and Pain Therapy master degree programs (which were taught with the same characteristics at the two universities that participated in the study), that their participation was voluntary, and that they attended all the traditional workshops or MAES© simulation sessions programmed. The participants were randomly assigned to the experimental group (*n* = 57), or the control group (*n* = 46) by using a random numbers table (once they accepted, their names were put on a list, and after assessing if they had similar profiles, a randomization table was utilized to assign them to either the experimental or the control group). This randomization was performed by the main researcher and by an administrative assistant from each university to avoid biases.

The study was conducted in the simulation spaces in both universities between October and November of 2018 and 2019. The collection of data took place in an assigned classroom at each university between November and July of 2019 and 2020.

All the students admitted to the master’s program had to have a similar profile for admittance, so it was implied that the samples from both groups were equivalent; also, the assignment to the different groups was random, as previously stated. Once the groups were defined, the participants were informed about the group they were to be part of on the first day of the intervention, and the control group was offered the opportunity to perform the intervention once the study ended.

### 2.3. Procedure

**Figure 1:**
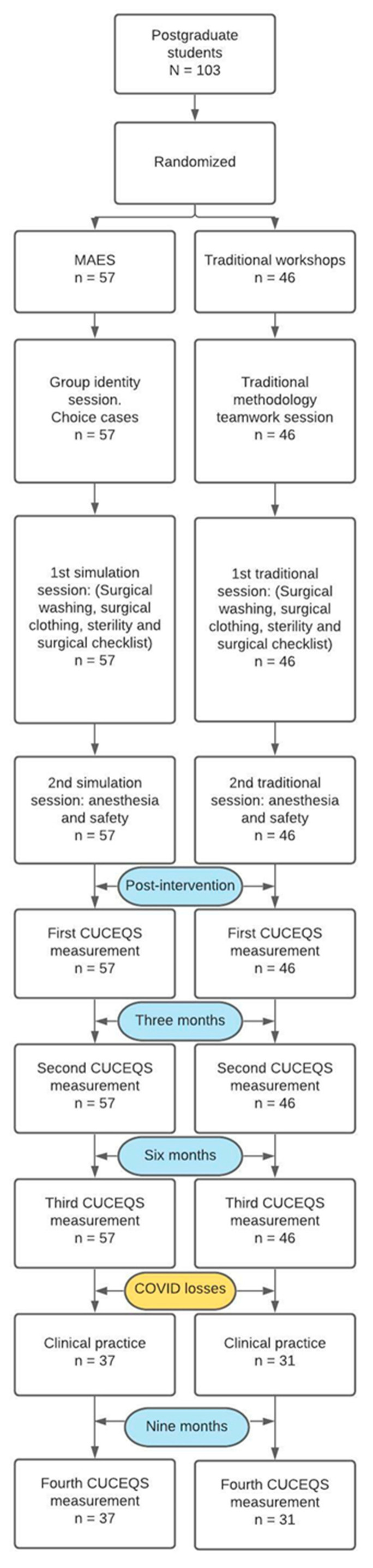
Structure of the study.

All the students took part in previous sessions about the general features of an operating room, the competencies of a surgical or perioperative nurse throughout the perioperative process, and the safety of the surgical patient. These prior sessions were identical for both the experimental and the control groups. The structure of the study is shown as a flow diagram (Figure 1).

#### 2.3.1. Experimental Group (Learning with MAES©)

Three sessions were conducted, which are described below:

- Session 1 (in-person).

The first 3 elements of the MAES© methodology are addressed in [20], which consist of the following: the creation of independent work teams, the selection of competencies/learning objectives, and the creation of an adequate work environment.

In the first MAES© session, group dynamics were implemented to select student teams with a defined identity and the establishment of a psychologically safe environment. In this same session, the students voluntarily selected the situations that their group would conduct research on, and a brainstorming session took place to establish the competencies and skills that would be the basis of their learning objectives. Each team chose a case that would serve as the basis for the design of a simulation scenario for the posterior in-person session.

The base level of the competences (prior knowledge of the participants) was detected in this phase through the brainstorming technique between the group members, who described what they knew or did not know about a specific subject selected by them among a series of real-life situations proposed by the facilitator (Table 1). In this session, a discussion was started until arriving at a consensus about the lack of knowledge or skills of the group with respect to a specific health problem that was associated with the safety of the surgical patient, which could be later addressed by the group.

The session ended with the commitment from all the teams to design a simulation scenario in accordance with a template that was explained by the professor, and which was available on the Internet: (https://www.youtube.com/watch?v=_7-6h0ZoD8k&feature=emb_logo (Access date: 2 November 2018; 13 November 2019).

From this point on, the teams worked on the design of a scenario that the other teams had to experience, and which would provide an answer to the learning objectives that the entire group had decided on through the brainstorming session on the selected situation.

- Sessions 2 and 3 (in-person).

The experimental group addressed the same areas of knowledge as the control group through two high-fidelity simulation sessions using the MAES© methodology. Each session lasted 4 h. In Sessions 2 and 3, simulation scenarios were developed, in which the students worked on surgical scrubbing, sterile surgical clothing in the operating room, the surgical instruments, sterility, and the use of the surgical checklist. In Session 3, 3 simulation scenarios were developed, in which the participants worked on anesthesia and safety throughout the perioperative process (Table 2).

In both sessions (2 and 3), the clinical simulation was performed by a team that was different from the one that designed the case. The team that designed the case was responsible for the set-up and soliciting of the materials needed from the person responsible for the simulation at the center. The rest of the participants, upholding the teams created, could participate in the simulation case created. Assignment to the case was random, so each group was exposed to each case at least one time.

After the simulation experience, in all the scenarios, a guided reflection session was led by the facilitator to discuss the simulation experience through the use of structured debriefing. Each of the teams that designed the scenario presented the evidence they found on the learning objectives proposed in the first session. The students had to provide high-quality scientific evidence about the subject matters addressed in the simulation, and the facilitator knew about the cases because he or she had checked them beforehand and had also verified the evidence.

#### 2.3.2. Control Group (Learning with Traditional Practical Seminars of Clinical Skills)

- Session 1 (in-person).

The control group received a traditional theoretical class about teamwork in the surgical environment.

- Sessions 2 and 3.

During the two posterior weeks, the control group took part in the two traditional theoretical–practical workshops, with sessions that lasted 4 h each. These sessions were based on a prior demonstration by the instructor and a posterior practice by the students. In one of the sessions, the students worked on surgical scrubbing, sterile surgical clothing in the operating room, the surgical instruments, sterility, and the use of the surgical checklist. In the other session, they worked on anesthesia and safety throughout the perioperative process.

### 2.4. Instrument and Data Collection

The tool utilized for the collection of data was the CUCEQS© questionnaire (the Spanish acronym of “Cuestionario de Competencias de la Enfermeria Quirurgica en Seguridad”). This questionnaire allows health professionals themselves to identify the dimensions of clinical safety they master, and those they have to improve to provide safe and high-quality perioperative care.

CUCEQS© is composed of 164 items divided into 4 theoretical competences and 17 sub-competences (Table 3). It is scored with a Likert-type scale from 0 to 4, with 0 being “not applicable”; 1 “very few times”; 2 “sometimes”; 3 “normally”; and 4 “always”.

The questionnaire has very good psychometric properties (with respect to internal consistency, it had excellent global reliability, with a Stratified Cronbach’s Alpha of 0.99), and excellent reliability according to competence (Cronbach’s Alpha for competences 1, 2, 3, and 4 of 0.80, 0.98, 0.94, and 0.93, respectively). As for the temporal stability of the questionnaire through the test–retest for the competences and sub-competences, the Intraclass Correlation Index was calculated, with a value higher than 0.77 obtained for all the cases, which points to the good reliability of the instrument. The tool designed also has high criteria and construct validities.

### 2.5. Data Analysis

The data were analyzed with the SPSS© v21 program (Statistical Package for the Social Sciences, IBM Corp. Released 2012). The statistical significance was set at 5%.

A descriptive analysis of the study variables was performed. For the quantitative variables, descriptive statistics, such as the mean and standard deviation were used, with frequencies and percentages used for the categorical values.

The comparison of the intra-subject scores in the different moments in time was performed through the use of Student’s *t*-test with the Bonferroni correction for multiple comparisons.

To calculate the inter-subject differences according to the learning methodology (learning with traditional practical seminars on clinical skills vs. learning with MAES©), a repeated measure, two-factor ANOVA (2 × 4) was performed for the competencies in surgical safety. The influence of the covariable of sex was controlled for, as it was not comparable in the baseline between the participants who took part in the training program. The assumption of this test is the condition of sphericity. However, when working with repeated measures, sphericity becomes the exception rather than the rule. Thus, in the case of not complying with the assumption, a multivariate approximation was utilized, which does not require that the matrix of variances–covariances be spherical, or the F critical value with the degrees of freedom modified through the correction index ε (Greenhouse–Geisser estimation). The effect size was calculated with eta square (*η2*), and to interpret it, we utilized the values of 0.01, 0.06, and 0.14, which indicate small, medium, and large effect sizes, respectively [25].

### 2.6. Ethical Considerations

The study was approved by the Ethics Committee of Research from the two universities where the study took place (protocol code: INF-2017-02; date of approval: 7 September 2017).

The participants were informed on the first day of the postgraduate course about the study and the benefits they could obtain at the level of training and the level of patient safety. In this session, their voluntary participation was asked of them, and those who wanted to participate signed the informed consent and the transfer of image rights forms. All the enrolled participants consented to participate in the study.

## 3. Results

### 3.1. Description of the Sample

Table 4 shows the sociodemographic and professional characteristics of the participants at baseline, as well as the differences of the group according to the learning methodology utilized. The sample was composed of 103 nurses at baseline, of which 46 (44.70%) participated in the traditional practical seminars of clinical skills, and 57 (55.30%) participated in the training based on the MAES© methodology. Their ages ranged from 22 to 55 years old (M = 31.12; SD = 7.46), with 80.60% of them women. The years of professional experience ranged from 0 to 25 years (M = 5.76; SD = 6.08). However, the experience in the operating room oscillated between 0 and 18 years (M = 0.98; SD = 2.51). In addition, 100% of the participants indicated having received training after their nursing degree. Thus, 45.6% had finished postgraduate courses, 36.9% had a master’s degree, 1.9% had a Ph.D., and 15.5% had other credentials.

### 3.2. Competencies in Surgical Safety

Table 5 and Table 6 show the results of the surgical safety competencies in the four measurements in both groups at the intragroup level.

With respect to the traditional practical seminar of clinical skills, decreases in the scores of the four competencies of surgical safety were observed, with these differences being statistically significant between the post-intervention measurement (nine months) and after 6 months, and between the measurements after 3 and 6 months (Table 5).

As for the group that participated in the learning with the MAES© method, just as the control group, decreases were observed in the scores of the four competencies of surgical safety, without significant differences found, in general, between the different measurements (Table 6).

Table 7 shows the results of the effects of the interaction of the time or moment of measurement by groups, controlling for the influence of the covariable of sex of the four competencies in surgical safety.

**Figure 2:**
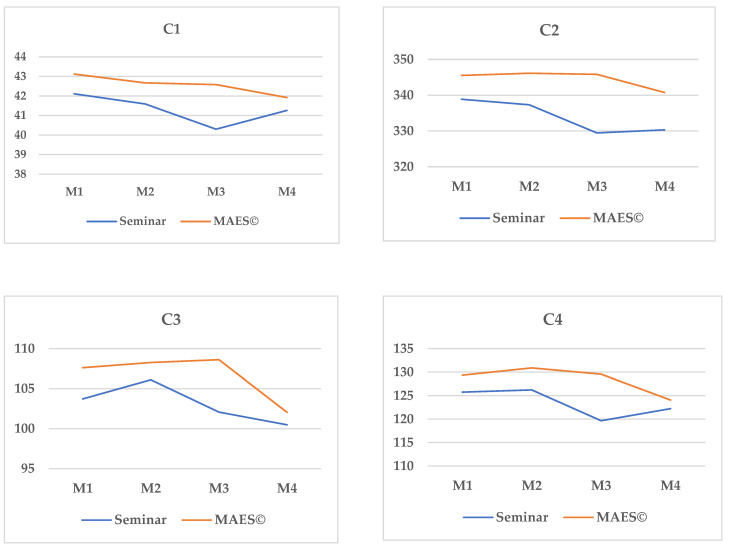
Pair-wise comparison of the measurements of the groups who took part in training through traditional practical clinical seminars on clinical skills and learning with MAES©. M = Measure; **C1** = exerts according to the legislation, ethics, and the professional orientation within the area of perioperative nursing; **C2** = provides perioperative nursing care integrating knowledge and evidence-based practice in a safe environment; **C3** = establishes and maintains effective interpersonal relationships with the users and the surgical team during the perioperative process; **C4** = promotes a culture of surgical patient safety; M1: first measurement (post-intervention, right after the intervention); M2: second measurement (3 months); M3: third measurement (6 months); M4: fourth measurement (9 months).

The results of the effect of the interaction in the four competencies in surgical safety show statistically significant differences (*p* < 0.001), which indicates that the changing trends in the scores after the training program were different in both groups; they were greater in the group that trained with the MAES© learning method, with a large effect size (Table 7 and Figure 2).

## 4. Discussion

The learning of competencies in surgical safety was more stable and superior in the experimental group that learned with MAES©. At every point in time when measurements were taken, the mean score of the evaluation tool was higher in the experimental group, and this difference was statistically significant with respect to the control group. Another interesting finding was obtained when comparing the intragroup scores at the different points in time. In both groups (experimental and control), the level of competence decreased with time (effect of forgetfulness), but in the experimental group, the decrease was not as accentuated as in the control group, as shown in Figure 2, without statistically significant differences found in the loss of the MAES© intragroup competence, although statistically significant differences were found in the control group.

Figure 2 shows that in both groups (experimental and control), the level of competence decreased with time, with the greatest decreased in Measurement 3 (M3–6 months). This effect has also been observed in other studies that assessed the acquisition of competencies, evidencing the loss of the degree of the competence acquired after 6 months [26]. On the other hand, it should be underlined that starting with Measurement 4 (M4), after the period of clinical practice, the measurements of the control group show an increased score since they incorporated what was learned into their clinical practice, while these scores decreased in the experimental group, mainly in the competencies associated with communication, leadership, teamwork, and culture of safety. This could be due to the fact that during the simulation, the students continuously worked in situations where these competencies were developed, while in their clinical practices, these were not developed in a standardized and meticulous manner, as shown by Urbach et al. in their study on the implementation of the surgical checklist used to promote a culture of safety in the surgical environment [27].

The score was higher and with a statistically significant difference in the experimental group in all the competencies, with a large size effect. These findings indicate that learning with MAES© was better utilized by the students and had greater stability over time than that obtained by seminars on perioperative nursing skills. In other studies, similar results were obtained in bachelor’s degree students when the MAES© method was compared with learning in non-self-directed simulations [21,22]. However, in this study, we can underline the improvement in the learning of postgraduate professionals, who theoretically possessed a greater degree of practical competence.

To interpret the results, we must refer to the current paradigm of teaching [28], which has changed with respect to the traditional paradigm to achieve higher quality learning. In this new paradigm, the students are given the leading role in their learning process and the acquisition of competencies is promoted, which results in a migration from the classic model of teaching to a multidimensional model that goes beyond the mere accumulation of knowledge. However, it is still possible to observe the great weight of the unidirectional learning methods on study plans, such as lecture-based classes with low involvement from the students. Little by little, learning methods in which the student has greater responsibility and independence (such as in the case of the MAES© method) are being successfully implemented, but the road to travel is long. In the case of nursing, high-fidelity clinical simulation has become highly important in the acquisition of skills [29], although the professor’s behavior still tends to lean towards a director’s role when deciding what the students must learn. What would happen if the students’ independence increased? Perhaps we can provide some answers to this question with the results of the study.

In the first place, we must discuss the concept of competence [30]. Competence is a dynamic construct composed of different dimensions (knowledge, skills, attitudes, aptitudes, and good judgment). For each of these dimensions, there is a training and evaluation system that is adapted to it, in which the teachers intervene, although their main motivation is to show that it is the students who must play an active role in their learning, beyond the direction of a specific study plan.

Simulation is a type of methodology that provides an almost immediate reflective answer to the behaviors of the students at the same time that they are being developed. This aspect stimulates the critical thinking of the students [31], especially when weaknesses are addressed in the simulated clinical practice. This improvement is relevant, as we believe that, in the end, it will affect the clinical safety of the surgical patient when the knowledge acquired in a simulation is transferred to real-life settings.

The application of the MAES© simulation in this study brings to light the direct relationship between the acquisition of competencies and the training phase. The results show the significant increase in the level of competence acquired by the students when having specific training on safety in a surgical setting through the use of a self-directed method, with respect to the students who took part in traditional workshops. We believe that one of the fundamental aspects to which we can credit the success of this method is the greater motivation of the students who learned in a self-directed manner. The importance of motivation for learning has been described before [32], and specifically, in a clinical simulation, this is a crucial aspect [33] that increases with the students’ autonomy.

Another explanation for these findings is that with MAES©, the students learn starting from a baseline of competences that they themselves establish [20]. It does not mean having to learn what was imposed by a study plan or what the professor believes they should learn, but, through group interaction dynamics, the students discuss the subject matter selected before the training session. Thus, we are able to discover what the students know or do not know about the subject before it is addressed. One basis of the MAES© methodology, as a learning method based on constructivist principles [34], is precisely the theory of significant learning by Ausubel [35], who postulated that true knowledge is attained if the new contents have meaning with respect to the previous knowledge possessed. The assimilation of new knowledge is achieved at the same time that the previous knowledge possessed becomes more stable and complete. Through time, both types of knowledge become fused into a single one, thereby increasing the overall quality of learning. This learning pushes aside memorization-based learning, in which new knowledge is forgotten easily, as it does not have any relationship with previous knowledge. As we have discussed, the starting point of the MAES© methodology is the previous knowledge of the participants, and it is developed as a function of what the students know and want to know about safety in an operating room environment. This is a critical aspect, as the long-term stability of the skills and knowledge of the nurses is crucial for considering the safety of the patients throughout the perioperative process.

Another important aspect is that the students design simulation scenarios and search for scientific evidence that is in line with the case. This manner of using knowledge promotes the creation of reflective professionals who base their practices on the available evidence. Thus, this also promotes evidence-based nursing practices (the students do not uncritically absorb the content in a course, but create knowledge starting with sources that they themselves search for and are shared with the group). Evidence-based practice [36] promoted by MAES© is a future guarantee of success, and an element that promotes the safety of the patient and the professional development of nurses.

We do not intend to diminish the importance of lecture-based traditional learning seminars, in which the role of the student is more passive. We believe that this method can be adequate as long as the basic level of the student’s competence is low so that they need to be closely guided. At the postgraduate level, the use of methods that promote the active involvement of the students, such as MAES©, is recommended. We believe that if surgical nursing professionals are trained to be more competent in the safety of the patient, it is probable that their behavior in their profession will also be more competent.

## 5. Conclusions

Postgraduate nurses are more competent in the safety of a surgical patient when they are trained with the MAES© methodology than when they learn through traditional theoretical–practical workshops.

The learning of competencies in surgical safety was more stable and superior in the experimental group who learned with MAES© than the control group.

Self-directed simulation learning is an adequate method for the acquisition of competencies, as it is based on experiential, reflective, and meaningful learning without risks to the patients. MAES© promotes autonomy and evidence-based practice in nursing.

This research study provides evidence in favor of training with MAES© as a differential methodology for the acquisition of perioperative nursing competencies associated with the safety of the surgical patient.

## 6. Strengths, Limitations, and Areas for Further Research

As the limitations, it should be mentioned that the research team was able to collect all the questionnaires in the moments in time measured, but some losses occurred in the 4th measurement in the 2019–2020 academic year due to the COVID-19 pandemic, as clinical practices were limited in Spain.

Data was not collected in time period 0, meaning a pretest, before the start of the academic year. This would have allowed us to discover the surgery safety competencies of the participants before they were assigned to the experimental and control groups. Nevertheless, as the participants had similar profiles when they were accepted into the postgraduate program, we consider that the groups were equivalent. We believe that a first measurement (pretest) would have had effects on the learning of surgical safety, and we did not want to consider this threat to the validity of the study.

To avoid biases, the main researcher was present in the development of the research study in both of the universities where the study took place.

## Figures and Tables

**Table 1:** Situations proposed and learning objectives.

Situation Proposed	Knowledge and Skills Chosen by the Group to Conduct Research On
Perioperative nurse https://www.youtube.com/watch?v=uBMdgprsmSA (Access date: 2 and 13 November 2018; 13 and 20 November 2019)	Knowledge of surgical scrubbing and sterile surgical clothing. Development of skills.
The ring https://www.youtube.com/watch?v=OgfJhOKmXog (Access date: 2 and 13 November 2018; 13 and 20 November 2019)	Knowledge of the correct putting on of gloves through the identification of an error. Development of skills.
Emergency https://www.youtube.com/watch?v=naSKwEYVfOM (Access date: 2 and 13 November 2018; 13 and 20 November 2019)	Surgical checklist (phases, development, nurse leadership, and multidisciplinary communication). Practice of WHO standards.
The mistake https://www.youtube.com/watch?v=HHD8mNhwG_I (Access date: 2 and 13 November 2018; 13 and 20 November 2019)	Learning about an error. Erroneous surgery. Practice of WHO standards.
News https://www.youtube.com/watch?v=_dKxcaTKDnE (Access date: 2 and 13 November 2018; 13 and 20 November 2019)	Learning about an error. Ethical problem when facing the death of a patient in surgery.
Awake https://www.youtube.com/watch?v=CTFEz2RPsWE (Access date: 2 and 13 November 2018; 13 and 20 November 2019)	Learning about the care and monitoring of a patient who receives general anesthesia. Development of the necessary skills.
They ruined his life https://www.youtube.com/watch?v=WxVTWOt0h00 (Access date: 2 and 13 November 2018; 13 and 20 November 2019)	Learning through different mistakes in surgery or anesthesia. Development of skills after the identification of a drug-related allergy.
How do you know if you are tired? https://www.youtube.com/watch?v=rK2Yh2GXE78 (Access date: 2 and 13 November 2018; 13 and 20 November 2019)	Learning about the importance of coordination and teamwork in an operating room.
**Communication/respect** https://www.youtube.com/watch?v=HS-Johhbl0s (Access date: 2 and 13 November 2018; 13 and 20 November 2019)	Development of the importance of the confidentiality of the patient and respect of intimacy.
**Communication/respect** https://www.youtube.com/watch?v=BSMF6JhW0tE (Access date: 2 and 13 November 2018; 13 and 20 November 2019)	Development of the importance of the confidentiality of the patient and respect of intimacy.

The design of the scenarios was an out-of-classroom task, performed with the advice of the facilitator at all times.

**Table 2:** Summary of the MAES© simulation scenarios.

Session 1 (2 h)	Session 2 (4 h)	Session 3 (4 h)
Prebriefing	Briefing (10′) Simulation experience (20′) Debriefing (30–35′)	Briefing (10′) Simulation experience (20′) Debriefing (30–35′)
Creation of simulation groups	Case 1 Simulation (1 h): Development of safe and quality care of perioperative nursing	Case 4 Simulation (1 h): Error/Perioperative nursing care related to general anesthesia
Presentation of learning objectives according to competencies. Selection of cases.	Case 2 Simulation (1 h): Surgery errors/ Surgical checklist	Case 5 Simulation (1 h): Perioperative nurse/Safe general anesthesia.
Definition of learning interests by the students.	Case 3 Simulation (1 h): Teamwork/Efficient communication/Nurse leadership in the perioperative process.	Case 6 Simulation (1 h): Error in postoperative analgesia administration/Pharmacological allergy.

In the simulation scenarios, high-fidelity simulators, an actor or standardized patient, were utilized. The research team considered that the union of these resources represented the best didactic content related to the security of the surgical patient.

**Table 3:** Competences and sub-competences of the CUCEQS© questionnaire.

Competence	Sub-Competence
1. EXERTS ACCORDING TO THE LEGISLATION, ETHICS, AND THE PROFESSIONAL ORIENTATION WITHIN THE AREA OF PERIOPERATIVE NURSING.	1.1 Ability to comply with safety standards. 1.2 Ability to apply the surgical verification list or surgical checklist
2. PROVIDES PERIOPERATIVE NURSING CARE INTEGRATING KNOWLEDGE AND EVIDENCE-BASED PRACTICE	2.1 Intervention as an anesthesia nurse related to the anesthesia technique. 2.2 Intervention as an anesthesia or circulating nurse in relation to the safety in the placement of the patient. 2.3 Intervention as an anesthesia or circulating nurse in relation to the surgical thermoregulation of the patient. 2.4 Intervention as a circulating nurse. 2.5 Intervention as a scrub nurse. 2.6 Intervention as an anesthesia or circulating or scrub nurse in relation to the use of the electric scalpel. 2.7 Intervention as a post-anesthetic care unit nurse. 2.8 Intervention as an anesthesia or circulating or post-anesthetic care unit nurse in relation to pain.
3. ESTABLISHES AND MAINTAINS EFFECTIVE INTERPERSONAL RELATIONSHIPS WITH THE USERS AND THE SURGICAL TEAM DURING THE PERIOPERATIVE PROCESS.	3.1 Ability to establish efficient communication with the user during the perioperative process. 3.2 Ability to develop strategies to promote communication and teamwork. 3.3 Ability of nurse leadership.
4. PROMOTES A CULTURE OF SURGICAL PATIENT SAFETY	4.1 Ability to promote a culture of patient safety centered on the professional. 4.2 Promotion of a culture of patient safety centered on the professional with respect to the organization or institution. 4.3 Ability to identify, notify, and communicate mistakes. 4.4 Ability of the nurse to develop scientific knowledge at each moment in the perioperative process.

**Table 4:** Sociodemographic, academic, and professional data of the participants in total and according to the learning methodology utilized.

	Seminars (*n* = 46)	MAES© (*n* = 57)	Total (*n* = 103)	Statistic Value *^a^*	*p*
Age (*M* (*SD*))	32.11 (7.63)	30.32 (7.28)	31.12 (7.46)	1.22	0.23
Sex (*n* (*%*))	-	-	-	4.16	**0.04**
Female	33 (71.70)	50 (87.70)	83 (80.60)	-	-
Male	13 (28.30)	7 (12.30)	20 (19.40)		-
Years of experience (*M* (*SD*))	6.09 (6.57)	5.49 (5.70)	5.76 (6.08)	0.49	0.62
Years of experience, operating room (*M* (*SD*))	0.78 (2.24)	1.14 (2.71)	0.98 (2.51)	−0.72	0.47
Posterior studies (%)	-	-	-	2.58	0.46
Masters	19 (41.30)	19 (30.30)	38 (36.90)	-	-
Postgraduate course	19 (41.30)	28 (49.10)	47 (45.60)	-	-
Doctorate	0 (0)	2 (3.50)	2 (1.90)	-	-
Others	8 (17.40)	8 (14.00)	16 (15.50)	-	-

Note: *^a^* = t for continuous quantitative values, and *X^2^* for qualitative variables; M = Mean; SD = Standard Deviation.

**Table 5:** Differences in means of the surgical safety competencies in the 4 measurements from the group who trained with the traditional seminars on practical clinical skills.

	M1	M2	M3	M4	*n* = 46		*n* = 46		*n* = 31		*n* = 46		*n* = 31		*n* = 31	
	*M (SD)*	*M (SD)*	*M (SD)*	*M (SD)*	*t*1	*p*	*t*2	*p*	*t*3	*p*	*t*4	*p*	*t*5	*p*	*t*6	*p*
C1	42.11	41.59	40.30	41.26	1.12	0.27	3.39	0.00	1.70	0.10	3.04	0.00	0.12	0.91	1.45	0.16
	(2.30)	(2.47)	(3.18)	(2.50)												
C2	338.87	337.33	329.46	330.32	0.52	0.61	2.93	0.01	1.66	0.11	2.62	0.01	0.93	0.36	0.00	1.00
	(13.75)	(18.52)	(18.86)	(28.23)												
C3	103.72	106.11	102.07	100.48	−1.45	0.15	1.12	0.27	0.85	0.40	3.56	0.00	1.83	0.08	0.57	0.57
	(10.95)	(9.45)	(8.81)	(14.01)												
C4	125.76	126.22	119.65	122.22	−0.25	0.81	2.95	0.01	1.25	0.22	4.23	0.00	0.71	0.48	−1.26	0.22
	(11.39)	(10.06)	(11.59)	(16.00)												

Note: M = mean; SD = standard deviation; C1 = exerts according to the legislation, ethics, and the professional orientation within the area of perioperative nursing; C2 = provides perioperative nursing care integrating knowledge and evidence-based practice in a safe environment; C3 = establishes and maintains effective interpersonal relationships with the users and the surgical team during the perioperative process; C4 = promotes a culture of surgical patient safety; M1: first measurement; M2: second measurement; M3: third measurement; M4: fourth measurement; *t*1 = differences between M1 and M2 measured with Student’s *t*-test; *t*2 = differences between M1 and M3 measured with Student’s *t*-test; *t*3 = differences between M1 and M4 months measured with Student’s *t*-test; *t*4 = differences between M2 and M3 months measured with Student’s *t*-test; *t*5 = differences between M2 and M4 months measured with Student’s *t*-test; *t*6 = differences between M3 and M4 months measured with Student’s *t*-test.

**Table 6:** Differences in means of the surgical safety competencies in the 4 measurements from the group who trained with the MAES© learning method.

	M1	M2	M3	M4	*n* = 57		*n* = 57		*n* = 37		*n* = 57		*n* = 37		*n* = 37	
	*M (SD)*	*M (SD)*	*M (SD)*	*M (SD)*	*t*1	*p*	*t*2	*p*	*t*3	*p*	*t*4	*p*	*t*5	*p*	*t*6	*p*
C1	43.12	42.67	42.58	41.92	1.65	0.10	1.86	0.07	2.67	0.01	0.28	0.78	0.57	0.57	1.07	0.29
	(1.46)	(1.95)	(2.33)	(2.66)												
C2	345.53	346.14	345.84	340.76	−0.27	0.79	−0.12	0.90	0.24	0.81	0.15	0.88	0.42	0.68	0.77	0.45
	(17.07)	(14.12)	(13.54)	(19.69)												
C3	107.61	108.26	108.61	102.05	−0.49	0.63	−0.83	0.41	1.03	0.31	−0.25	0.80	1.36	0.18	2.66	0.01
	(10.39)	(11.06)	(9.69)	(12.56)												
C4	129.33	130.88	129.56	124.06	−0.98	0.33	−0.17	0.86	0.32	0.75	0.95	0.35	1.18	0.25	1.18	0.25
	(11.08)	(10.92)	(10.44)	(14.36)												

Note: M = mean; SD = standard deviation; C1 = exerts according to the legislation, ethics, and the professional orientation within the area of perioperative nursing; C2 = provides perioperative nursing care integrating knowledge and evidence-based practice in a safe environment; C3 = establishes and maintains effective interpersonal relationships with the users and the surgical team during the perioperative process; C4 = promotes a culture of surgical patient safety; M1: first measurement; M2: second measurement; M3: third measurement; M4: fourth measurement; *t*1 = differences between M1 and M2 measured with Student’s *t*-test; *t*2 = differences between M1 and M3 measured with Student’s *t*-test; *t*3 = differences between M1 and M4 months measured with Student’s *t*-test; *t*4 = differences between M2 and M3 months measured with Student’s *t*-test; *t*5 = differences between M2 and M4 months measured with Student’s *t*-test; *t*6 = differences between M3 and M4 months measured with Student’s *t*-test.

**Table 7:** Differences between inter-subject means.

	M1		M2		M3		M4		Intergroup A × B		
	*M*	*(SD)*	*M*	*(SD)*	*M*	*(SD)*	*M*	*(SD)*	*F*	*p*	*η2*
**C1**											
Seminar (*n* = 31)	42.11	(2.30)	41.59	(2.47)	40.30	(3.18)	41.26	(2.50)	3311.57	<0.001	0.98
MAES© (*n* = 37)	43.12	(1.46)	42.67	(1.95)	42.58	(2.33)	41.92	(2.66)			
**C2**											
Seminar (*n* = 31)	338.87	(13.75)	337.33	(18.52)	329.46	(18.86)	330.32	(28.23)	4225.01	<0.001	0.99
MAES© (*n* = 37)	345.53	(17.07)	346.14	(14.12)	345.84	(13.54)	340.76	(19.69)			
**C3**											
Seminar (*n* = 31)	103.72	(10.95)	106.11	(9.45)	102.07	(8.81)	100.48	(14.01)	898.18	<0.001	0.93
MAES© (*n* = 37)	107.61	(10.39)	108.26	(11.06)	108.61	(9.69)	102.05	(12.56)			
**C4**											
Seminar (*n* = 31)	125.76	(11.39)	126.22	(10.06)	119.65	(11.59)	122.22	(16.00)	949.62	<0.001	0.94
MAES© (*n* = 37)	129.33	(11.08)	130.88	(10.92)	129.56	(10.44)	124.06	(14.36)			

M = Mean; SD = Standard Deviation; η2 = eta square; C1 = exerts according to the legislation, ethics, and the professional orientation within the area of perioperative nursing; C2 = provides perioperative nursing care integrating knowledge and evidence-based practice in a safe environment; C3 = establishes and maintains effective interpersonal relationships with the users and the surgical team during the perioperative process; C4 = promotes a culture of surgical patient safety; M1: first measurement; M2: second measurement; M3: third measurement; M4: fourth measurement.

## Data Availability

Data are available by contacting the corresponding authors.
